# Serious hemorrhages after ischemic stroke or TIA – Incidence, mortality, and predictors

**DOI:** 10.1371/journal.pone.0195324

**Published:** 2018-04-05

**Authors:** Joachim Ögren, Anna-Lotta Irewall, Lars Söderström, Thomas Mooe

**Affiliations:** 1 Department of Public Health and Clinical Medicine, Östersund, Umeå University, Sweden; 2 Unit of Research, Development and Education, Östersund, Sweden; Universitatsklinikum Freiburg, GERMANY

## Abstract

**Background:**

Data are lacking on the risk and impact of a serious hemorrhage on the prognosis after ischemic stroke (IS) or transient ischemic attack (TIA). We aimed to estimate the incidence of serious hemorrhage, analyze the impact on mortality, and identify predictors of hemorrhage after discharge from IS or TIA.

**Methods and findings:**

All patients admitted to Östersund Hospital for an IS or TIA in 2010–2013 were included (n = 1528, mean age: 75.1 years). Serious hemorrhages were identified until 31^st^ December 2015. Incidence rates were calculated. The impact on mortality (stratified by functional level) was determined with Kaplan-Meier analysis. Non-parametric estimation under the assumption of competing risk was performed to assess the cumulative incidence and predictors of serious hemorrhages.

The incidence rates of serious (n = 113) and intracranial hemorrhages (n = 45) after discharge from IS and TIA were 2.48% and 0.96% per year at risk, respectively. Patients with modified Rankin Scale (mRS) scores of 3–5 exhibited 58.9% mortality during follow-up and those with mRS scores of 0–2 exhibited 18.4% mortality. A serious hemorrhage did not affect mortality in patients with impaired functional status, but it increased the risk of death in patients with mRS scores of 0–2. Hypertension was associated with increased risk of serious hemorrhage.

**Conclusions:**

We found that, after discharge from an IS or TIA, serious hemorrhages were fairly common. Impairments in function were associated with high mortality, but serious hemorrhages only increased the risk of mortality in patients with no or slight disability. Improved hypertension treatment may decrease the risk of serious hemorrhage, but in patients with low functional status, poor survival makes secondary prevention challenging.

## Introduction

An ischemic stroke (IS) or transient ischemic attack (TIA) is associated with increased risk of a new ischemic stroke, death, and other cardiovascular events[1–3]. To decrease the risk of new ischemic events, a majority of patients are discharged with antithrombotic treatment. The preventive effect of antithrombotic treatments has been shown in randomized controlled trials (RCT) [4–10], but these treatments were also associated with the risk of serious hemorrhage, a potentially devastating complication. The risk of serious hemorrhage in patients treated with the anticoagulant (AC), warfarin, varied between 2–5% per year, and the risk in patients treated with antiplatelets (AP) varied between 1–2% per year [8–16]. The risk of causing a serious hemorrhage must be considered when prescribing AC or AP after IS or TIA, but there is limited knowledge of how common this potentially devastating complication is in clinical practice. Only a few cases of hemorrhages have been reported in population-based cohort studies [17–19]. Furthermore, the populations included in RCTs are often younger and have less comorbidity than the average patient treated in a stroke unit. Because these factors might affect the incidence of hemorrhage, uncertainty has arisen over currently estimated incidences of hemorrhage in a general population of patients with strokes and TIAs. In a national-level registry study from Sweden, with a mean age approximately 10 years older than the average age reported in the RCTs, the incidences of post-stroke hemorrhage in patients treated with AC and AP were 2.5% and 2.4% per year at risk, respectively[20]. In the same study, the risk of hemorrhage in patients without any antithrombotic treatment was higher than in patients treated with AC or AP. That finding suggested that the untreated group had a high prevalence of other diseases and, perhaps, low functional status. It is known that low functional status after a stroke predicts worse survival [21], but it is unknown how survival might be impacted by a hemorrhage in relation to the level of functional status. Registry studies typically lack the option of validating diagnoses. Therefore, data are needed from an unselected population, outside the RCTs, that offer the possibility of scrutinizing patient records and validating data. The present study aimed: (1) to determine the incidence of validated serious hemorrhages and survival, after discharge, in an unselected cohort of patients that were hospitalized with IS and TIA; (2) to determine the incidence of a serious hemorrhage in relation to the functional level at discharge, and the impact on survival; and (3) to identify factors associated with increased or decreased risk of hemorrhage.

## Material and methods

### Study design and setting

In this population-based cohort study, we included all patients in the registry of the Nurse-based Age-independent Intervention to Limit Evolution of Disease (NAILED) stroke trial [22]. The registry contained all patients admitted to Östersund Hospital with a stroke or TIA, between January 1^st^, 2010 and December 31^st^, 2013. The hospital of Östersund is the only hospital in the county of Jämtland, a geographically large, rural area in central Sweden with approximately 125,000 inhabitants. With the exception of patients in terminal care, all patients with strokes and TIAs in the county are referred to the hospital. During the screening phase of the NAILED trial, hospital records of all patients that had undergone computed tomography (CT) brain scans were reviewed daily to identify patients that were subsequently diagnosed with an acute stroke or TIA. Mechanisms of serious hemorrhage can differ between the acute phase and the more stable follow-up period after discharge (e.g. fibrinolytic treatment and hemorrhagic transformation of an ischemic stroke increase the incidence of serious hemorrhage in-hospital. Also, the risk of gastrointestinal (GI) hemorrhage is higher during the acute phase). In the present study we focused on the risk of hemorrhage during long-term follow-up after discharge. Out of 1607 identified patients, 72 died in hospital and 7 had a serious hemorrhage prior to discharge (of which none were fatal); those patients were excluded from this study (see S1 and S2 Figs).

### Data collection and outcomes

A serious hemorrhage was defined as a hemorrhage that required admission to the hospital. It was classified as fatal when the patient died within 30 days. All hemorrhages were classified as intracranial (ICrH), gastrointestinal (GI), or “others”. To identify serious hemorrhage events, we searched the local database of discharge diagnoses to identify any contact with the hospital due to a hemorrhage. The ICD-10 codes for hemorrhage (S1 Table) were included in the search. An identified event was then validated against the patient records, and it was included in the study when (1) an ICrH was diagnosed, (2) a hemorrhage was the main cause of admission, or (3) the hemorrhage required transfusion or surgery. When a patient had more than one serious hemorrhage, only the first was included in this study.

Patients with IS or TIA were followed from the day of discharge from hospital to an event considered a serious hemorrhage, a death, a move away from the county, or December 31^st^ 2015, whichever occurred first.

Data regarding risk factors and medication were collected from the NAILED database. During the study period, AC treatment was synonymous with warfarin administration. AP treatment was either clopidogrel, 75 mg once daily, acetylsalicylic acid (ASA), 75 mg once daily, or a combination of ASA (75 mg once daily) and dipyridamol (200 mg twice daily). Diabetes mellitus included both type I and type II diabetes. A smoker was defined as an individual with a current smoking habit or an individual that had ceased a smoking habit within the last 6 months. The patient’s functional level was categorized according to the modified Rankin Scale (mRS), where 0 indicated no symptoms, and 5 indicated severe disability (mRS scale, see S2 Table). Functional levels were assessed during hospitalization for the index event. For analyses according to functional level, the patients were divided into two groups: those with mRS scores of 0–2 and those with mRS scores of 3–5. A mRS score of 0–2 indicated that the patient could live independently, and a mRS score of 3–5 indicated significant disability which has previously been associated with poor survival [21].

### Statistical methods

Incidence rates were calculated to describe the incidence of serious hemorrhages per year at risk. We performed non-parametric estimation under the assumption of competing risk to assess the cumulative incidence of serious hemorrhage after discharge and Gray’s test for equality of cumulative incidence functions was used for group comparisons. The 30-day case fatality rates were calculated stratified for subtype of hemorrhage. Moreover, we performed Kaplan-Meier analyses to assess the survival after discharge, and the impact of a serious hemorrhage on survival, according to a high or low functional level, defined by the two mRS groups. The log-rank test was used for group comparisons. Risk factors were estimated using the method of Fine and Gray for competing risk. Possible risk factors for hemorrhage were assessed in univariable Cox regression analyses, and variables with a p-value <0.10, age, and sex were included in a multivariable model. We reduced the model stepwise by excluding the least significant variable manually until only significant variables remained. Gender and age were retained regardless of significance. The final multivariate analysis was based on complete cases with no missing data. Results are presented as hazard ratios (HRs) with a 95% confidence interval (95% CI). P-values <0.05 were considered significant. Statistical analyses were performed with IBM SPSS v.24 and SAS v.9.4

### Ethics

This study was approved by the Regional Ethics Committee in Umeå, on October 28, 2009 (Dnr: 09-142M), with supplements on June 10, 2013 (Dnr: 2013-204-32M) and January 13, 2015 (Dnr: 2014-416-32M).

## Results

**Fig 1:**
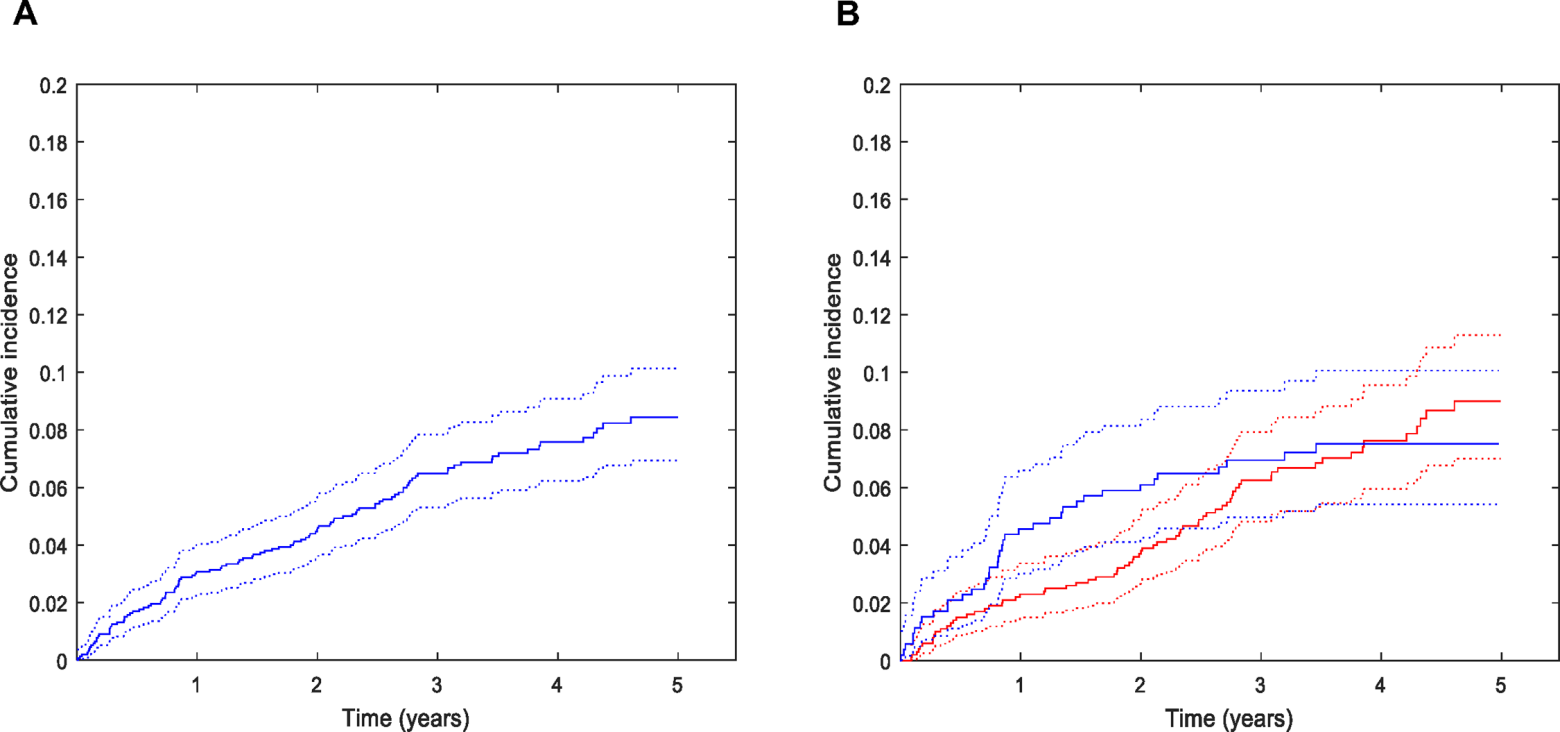
Cumulative incidence of serious hemorrhage five years after ischemic stroke and transient ischemic attack with 95% confidence interval. (A) All patients n = 1528, serious hemorrhages, n = 111. Two patients had a serious hemorrhage after five years from discharge. (B) Patients stratified by functional status. mRS: modified Rankin Scale.

A total of 1528 stroke and TIA patients was followed from discharge for a total observed person-time of 1622454 days (4445 years), median observed time 1099 days (interquartile range (IQR): 734–1556) Background data of the patients are shown in Tables 1 and S3. Study flow-charts are shown in S1 and S2 Figs. During follow-up, 125 patients had at least one hospitalization that included hemorrhage in the discharge diagnosis (of these, 27 had hemorrhage as a secondary diagnosis). Twelve patients, including two with hemorrhage as a primary diagnosis, did not meet our criteria of a serious hemorrhage. Thus, 113 (7.4%) patients had a serious hemorrhage, corresponding to an incidence rate of 2.48% (95% CI: 2.05–2.97) per year at risk. The median time to the event was 535 days (IQR: 209–942). In the two functional groups, mRS 0–2 and mRS 3–5, the incidence rates of serious hemorrhage were 2.19% (95% CI: 1.74–2.74) and 3.30% (95% CI: 2.38–4.47) per year at risk, respectively. Cumulative incidences of hemorrhage over the study period and incidence rates in different patient categories are given in Fig 1, Tables 2 and 3. ICrH was the most common bleeding subtype (n = 45, 39.8%), followed by GI hemorrhage (n = 41, 36.3%), and finally, others (n = 27, 23.9%, mostly urinary tract hemorrhages).

**Table 1:** Characteristics of patients with ischemic stroke or transient ischemic attack.

Characteristics	All	Serious hemorrhage	
	**n (%) **	**n (%) No**	**n (%) Yes**
All patients, N	1528	1415	113
Female	681 (44.6)	636 (44.9)	45 (39.8)
Age, y (mean)	75.1	74.9	77.4
Smoker	191 (12.7)	179 (12.9)	12 (10.9)
GFR at index event, mL/min/1.73 m^2^ (mean)	74	74	66
Ischemic stroke as index event	1083 (70.9)	999 (70.6)	84 (74.3)
Diagnosis prior to IS or TIA			
Hypertension	985 (64.5)	899 (63.5)	86 (76.1)
Myocardial Infarction	182 (11.9)	165 (11.7)	17 (15.0)
Heart Failure	123 (8.0)	111 (7.8)	12 (10.7)
Ischemic stroke	235 (15.4)	217 (15.4)	18 (15.9)
ICrH	39 (2.6)	35 (2.5)	4 (3.5)
GI hemorrhage	104 (6.8)	93 (6.6)	11 (9.7)
Diabetes at discharge	305 (20.0)	283 (20.0)	22 (19.5)
Atrial fibrillation	391 (25.6)	358 (25.3)	33 (29.2)
Treatment at discharge			
Statins	951 (62.2)	887 (62.7)	64 (56.6)
Antihypertensives	1184 (77.5)	1090 (77.0)	94 (83.2)
Anticoagulants	244 (16.0)	224 (15.8)	20 (17.7)
Antiplatelets	1233 (80.7)	1144 (80.8)	89 (78.8)
CHA2DS2-Vasc at discharge (mean)	4.9	4.8	5.1
mRS at discharge (mean)	1.8	1.8	1.9
mRS 0–2	1002 (65.7)	928 (65.6)	74 (65.5)
mRS 3–5	526 (34.4)	487 (34.4)	39 (34.5)

Values represent numbers of patients, with percentage of patients in each group in parantheses, unless otherwise indicated.

GFR indicates glomerular filtration rate; IS, ischemic stroke; TIA, transient ischemic attack; ICrH, intracranial hemorrhage; GI, gastrointestinal and mRS, modified Rankin scale.

**Table 2:** Incidence rates of different types of serious hemorrhage after hospitalization for ischemic stroke or transient ischemic attack in different patient groups.

Patient groups	Total	ICrH	GI	Others	ICH
All patients	2.48 (113)	0.96 (45)	0.88 (41)	0.58 (27)	0.40 (19)
Patients with IS	2.72 (84)	1.07 (34)	0.94 (30)	0.63 (20)	0.50 (16)
Patients with TIA	1.98 (29)	0.74 (11)	0.74 (11)		
Patients discharged with AC	2.74 (20)	1.20 (9)	1.07 (8)		
Patients discharged with AP	2.37 (89)	0.93 (36)	0.78 (30)		
Patients with mRS 0–2	2.19 (74)	0.93 (32)	0.66 (23)		
Patients with mRS 3–5	3.31 (39)	1.06 (13)	1.48 (18)		

Values represent the percentage (number of patients) of persons with serious hemorrhage per year at risk in each group.

ICrH indicates intracranial hemorrhage; GI, gastrointestinal and ICH intracerebral hemorrhage. ICrH includes ICH.

**Table 3:** Cumulative incidence of different types of serious hemorrhage after hospitalization for ischemic stroke or transient ischemic attack.

Years	Total		ICrH		GI		Others	
	n	incidence % (95%CI)	n	incidence % (95%CI)	n	incidence % (95%CI)	n	incidence % (95%CI)
1	47	3.08 (2.30–4.04)	13	0.85 (4.80–1.42)	17	1.11 (0.68–1.75)	17	1.11 (0.68–1.75)
2	71	4.66 (3.68–5.80)	25	1.64 (1.09–2.38)	26	1.71 (1.14–2.46)	20	1.31 (0.83–1.98)
3	96	6.56 (5.36–7.91)	38	2.62 (1.88–3.54)	34	2.32 (1.64–3.19)	24	1.62 (1.07–2.37)
4	106	7.64 (6.29–9.15)	42	3.07 (2.23–4.20)	39	2.85 (2.05–3.86)	25	1.72 (1.14–2.49)
5	111	8.50 (6.99–1.02)	45	3.59 (2.60–4.82)	40	3.02 (2.17–4.09)	26	1.89 (1.24–2.76)

Values represent number (n) and percentage of persons with serious hemorrhage in each group.

CI indicates Confidence Interval, ICrH intracranial hemorrhage and GI gastrointestinal.

Of the 113 serious hemorrhages, 18 (15.9%) were fatal within 30 days. Among the three main hemorrhage subtypes, ICrHs were associated with the highest 30-day case fatality (24.4%), but ICH (Intracerebral hemorrhage) alone was associated with a case fatality of 42.1% within 30 days (S4 Table). Among patients with mRS 0–2, eleven had fatal hemorrhages and eight were ICrHs. Among patients with mRS 3–5, seven had fatal hemorrhages; three were ICrHs and three were GI hemorrhages.

**Fig 2:**
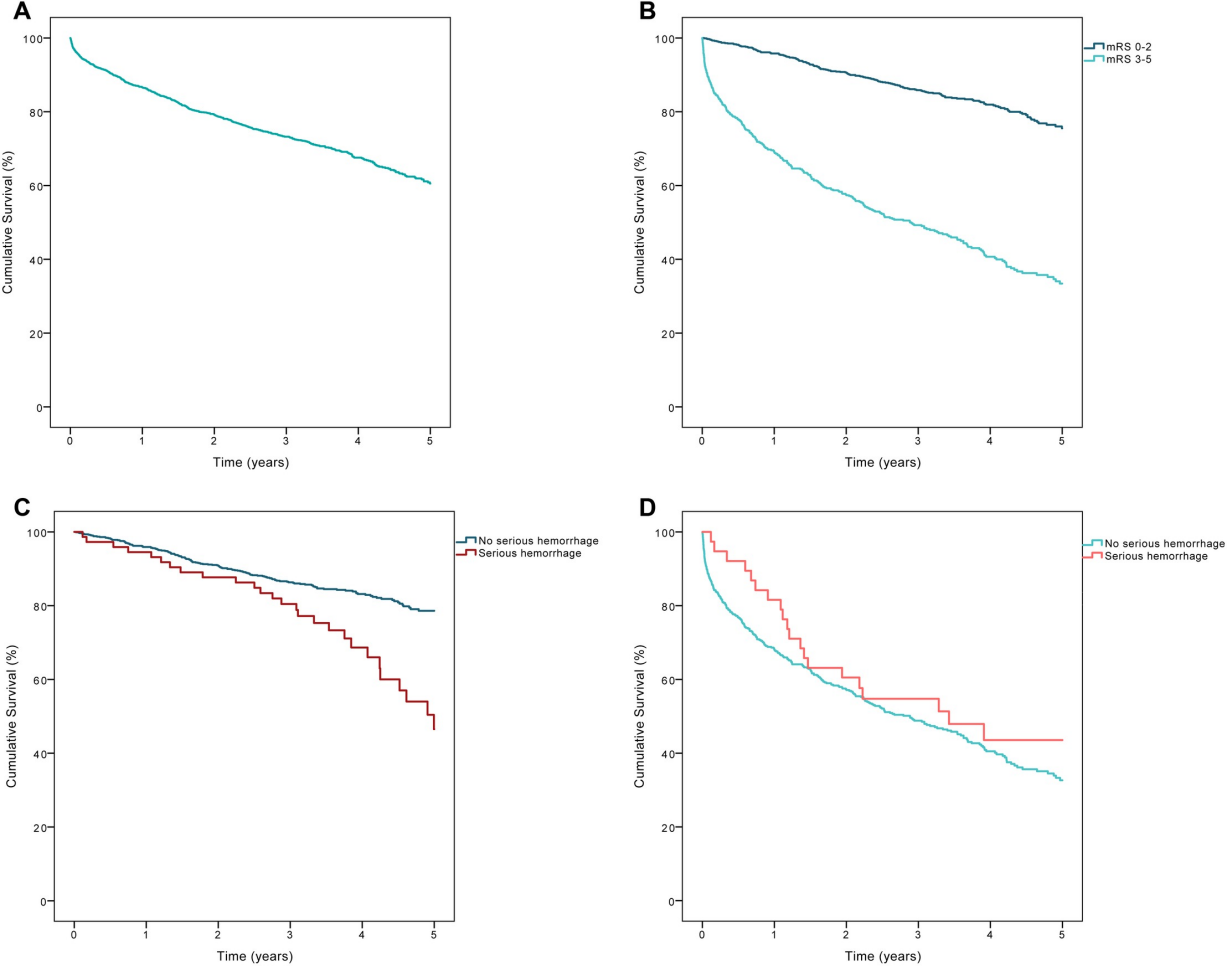
Cumulative survival five years after discharge from hospitalization for ischemic stroke or transient ischemic attack. (A) All patients (n = 1528, of which 485 died). (B) Patients stratified by functional level at discharge (p <0.001). (C) Patients with mRS 0–2 at discharge, with or without a serious hemorrhage during follow up (p <0.001). (D) Patients with mRS 3–5 at discharge, with or without a serious hemorrhage during follow up (p = 0.319); mRS: modified Rankin Scale.

A total of 494 (32.3%) participants died during follow-up, and of those, 205 (13.4%) died within the first year after discharge (Fig 2). Among participants with a functional level corresponding to mRS 0–2 at discharge, the mortality during follow-up was 18.4%, and the one-year mortality was 4.2%. In the subgroup with mRS 3–5, mortality during follow-up was 58.9%, and the one-year mortality was 31.0% (Fig 2). Among patients with mRS 0–2, a serious hemorrhage during follow-up was associated with higher mortality, compared to no hemorrhage (37.8% vs. 16.8%, p <0.001). However, among patients with mRS 3–5, a serious hemorrhage did not increase mortality, compared to no hemorrhage (53.8% vs. 59.3%, p = 0.319, Fig 2).

In the multivariable Cox regression analysis, hypertension prior to the IS or TIA was associated with increased risk of hemorrhage (Table 4). Treatment with AC or AP at discharge was not associated with increased risk of hemorrhage (see S5 Table for univariable and multivariable analysis without competing risk assumption).

**Table 4:** Multivariable cox regression analysis (final model) indicates predictors of the risk of serious hemorrhage, after ischemic stroke or transient ischemic attack (n = 1528).

Risk factors	HR (95% CI)	P-value
Age ≥75 years	1.30 (0.85–1.99)	0.220
Women	0.75 (0.52–1.09)	0.127
Prior Hypertension	1.72 (1.10–2.68)	0.017
Statin at discharge	0.81 (0.55–1.20)	0.300

HR indicates hazard ratio and CI confidence interval.

## Discussion

In this population-based cohort study, which included all patients with an IS or TIA diagnosis that were discharged from Östersund Hospital in 2010–2013, the incidence rates of serious hemorrhage and ICrH were 2.48% (95% CI: 2.05–2.97) and 0.96% (95% CI: 0.71–1.28) per year at risk, respectively. Patients with impaired functional status (mRS 3–5) at discharge had markedly higher mortality (58.9%) during follow-up compared to patients discharged with mRS 0–2 (18.4%). A serious hemorrhage did not affect the prognosis in patients discharged with mRS 3–5, but it increased the risk of death during follow-up in those with mRS 0–2. In our population, a diagnosis of hypertension was associated with an increased risk of serious hemorrhage.

The definition of a serious hemorrhage differs among studies [23–25], which complicates comparisons. We defined major bleeding simply as any bleeding that required hospital admission, as in other studies [16, 20]. We included events that required blood transfusion or surgery, even when the bleeding was not stated as the primary reason for admission.

### Incidence

This study determined the incidence rates of serious hemorrhages among unselected patients with previous stroke, both overall and in groups stratified by functional level. These data are useful in clinical situations, when it is important to take into account the risk of hemorrhage.

The incidence rates of 2.48% per year at risk, for all serious hemorrhages, and 2.72% in patients with IS were comparable to the incidence rate of 2.55% reported previously in an observational study based on patients with IS from the Swedish stroke-registry, Riksstroke [20]. In that study, the incidence rates for ICrH and GI hemorrhages were 0.89% and 1.18% per year at risk, respectively. The mean duration of follow-up was 3.0 years in our study, compared to 2.0 years in the Riksstroke study. As shown in Table 2, the cumulative incidence of GI and other hemorrhages (mostly urinary tract hemorrhages) was highest in the first post-stroke year, but the incidence of ICrH increased similarly each year. This result might explain the higher proportion of GI hemorrhages (46.8% vs. 36.2%) found in the registry study. Another study based on Riksstroke data included patients with a recent IS, and it focused on ICrH incidence [26]. Traumatic ICrH was not included in that study, but the incidence of ICH was 0.59% per year at risk, which was comparable to the incidence rate of 0.50% found in the present study.

In RCTs, the incidence of serious hemorrhage was reported to be higher in patients treated with AC than in patients treated with AP. Pooled data from 13 RCTs showed incidence rates of 2.5% and 1.0% per year at risk [16], respectively, which indicated that AP was associated with lower risk than AC. However, in both the present study and the Riksstroke study [20], the risk of hemorrhage differed only slightly between groups given these treatments, which suggested that AC and AP were associated with a similar risk of hemorrhage in unselected populations. Thus, we found incidence rates of 2.74% (95% CI: 1.72–4.16) and 2.37% (95% CI: 1.92–2.90) per year at risk in patients discharged with AC and AP. The discrepancy between our study and the RCTs might be explained by age differences. The patients in the RCTs had mean ages between 59 and 70 years, and the patients in our study had a mean age of 75 years. Some RCTs [5, 27, 28] as well as a population based cohort study [29] have found that increasing age was associated with a higher risk of AP-associated hemorrhage. This factor might at least partly explain the higher incidence rate found in our unselected population compared to that reported in RCTs.

We also found that the incidence of serious hemorrhage was higher among patients with mRS 3–5 than those with mRS 0–2 (Table 3). In the Cox regression analysis, impaired functional status was not independently associated with an increased risk of serious hemorrhage, but patients with mRS 3–5 were typically older and had more comorbidity than patients with mRS 0–2, which might explain the increased risk.

### The effect of a serious hemorrhage on survival

During follow up, 32.3% of the patients died. A previous study showed that the prognosis depended on functional status at 3 months [21]. In the present study, the assessment of functional level was based on status at discharge; therefore, some patients could have improved during the first months. Despite this limitation, our study showed that the status at discharge predicted survival. We observed a vast difference in survival between patients discharged with good function (mRS 0–2, 4.2% mortality) and those with impaired function (mRS 3–5, 31.0% mortality; Fig 2).

After a serious hemorrhage, the 30-day case fatality was 15.9% overall, and it was 24.4% in patients with an ICrH (S4 Table). In patients with mRS 0–2, most fatal hemorrhages (n = 8/11) were ICrHs, whereas, in patients with mRS 3–5, ICrH (n = 3/7) and GI hemorrhages (n = 3/7) were equally common. Although these subgroups are represented by very few cases, this indicates that ICrH was the most common fatal hemorrhage, but also that a fatal GI hemorrhage was more common in patients with impaired function. Although patients with good functional status showed a low all-cause mortality (4.2%) during the first year of follow-up, we found that a serious hemorrhage decreased survival during the long-term follow-up. However, among patients with impaired function (mRS 3–5), a serious hemorrhage did not worsen survival, probably due to the high mortality rate, irrespective of hemorrhage (Fig 2). It is unclear whether secondary preventive treatments might improve survival in a group of patients at high risk of mortality. On the other hand, we do not know whether the mortality rate might have been even higher without current secondary prevention or whether preventive treatments might have improved other aspects of well-being.

### Factors associated with increased risk of serious hemorrhage

In the multivariable Cox regression analysis, hypertension prior to the IS or TIA event was associated with an increased risk of serious hemorrhage. Prolonged hypertension affects the blood vessels, and it is a known risk factor for vascular disease, such as myocardial infarction and any type of stroke, including ICH. We studied an unselected, aged population; in this cohort, a high proportion of participants had had previous myocardial infarctions and/or strokes, and many had hypertension. An accumulation of risk factors, such as hypertension, has been associated with increased risk of hemorrhage [5, 30], and we found that an increased CHA2DS2-VASc score was associated with an increased risk of serious hemorrhage in the univariable Cox regression analysis (S5 Table). However, due to multicollinearity, the CHA2DS2-VASc score was not included in the multivariable model.

Statin treatment was not associated with an increased risk of serious hemorrhage (no association in competing risk model HR 0.81 (95% CI: 0.55–1.20, reduced risk without competing risk assumption HR 0.63 (95% CI: 0.43–0.93), see S5 Table). It is controversial whether statin therapy affects the risk of hemorrhage. Patients with recent IS or TIA showed a significant increase in the ICH occurrence in the statin trial, Stroke Prevention by Aggressive Reduction in Cholesterol Levels (SPARCL), [31] but the occurrence was insignificantly increased in the Heart Protection Study (HPS) [32]. In a previous large registry based study, there was no association between statin treatment and ICrH [33]. A few studies have examined the relationship between statins and the risk of GI hemorrhage, but the results were inconclusive [34–37]. Consequently, there is no clear association between statin treatment and serious hemorrhages among unselected stroke/TIA populations.

In the present study, we found that statins were given more often to relatively younger patients (<75 vs. ≥ 75 years) and patients with relatively higher functional levels (mRS 0–2 vs. mRS 3–5). However, we found no interaction between statins and mRS or between statins and age that affected the risk of serious hemorrhage.

We found that treatment with AP or AC at discharge was not independently associated with increased risk of hemorrhage, consistent with a previous study [20]. That finding could be related to either good clinical management or to the fact that patients discharged without treatment were the oldest and sometimes had complex comorbidities, which might have confounded the analyses.

### Strengths and limitations

This study included an unselected population. The cohort consisted of all patients in the target group discharged from Östersund Hospital during the study inclusion period. Our estimated risk of a serious hemorrhage after IS or TIA was based on a patient group with many older subjects. Older patients often have extensive co-morbidities, which is considerably different from patients included in clinical trials. However, this patient group reflected the characteristics of most patients in stroke units and according to the Swedish stroke quality register Riksstroke, the stroke care in Östersund was comparable to the rest of Sweden regarding age, diagnoses and secondary preventive treatment [38]. Thus, this feature of the study entailed good external validity. At the same time, unselected populations often contain subgroups with markedly different prognoses, as illustrated by the different functional groups in the present study. Subgroup characteristics must be considered before the results can be generalized. Unlike registry studies, we could verify outcomes against the medical records, which increased the internal validity of our study.

The study population was sufficiently large for estimating incidence rates and cumulative incidences. However, with 1528 patients and 113 serious hemorrhages, the Cox regression predictor analysis could only be exploratory. Consequently, although we could not confirm or dismiss any of the possible risk factors, we could explore whether any potential risk factor deviated in an unexpected direction.

This study was based on an observational cohort design. There is always the risk that confounders might not be taken into account, because they were not recorded as variables in the database. Furthermore, no conclusions concerning causality could be drawn.

## Conclusions

Among patients discharged after hospitalization for IS or TIA, the incidences of serious hemorrhage and ICrH were approximately 2.5% and 1.0% per year at risk, respectively. The 30-day case fatality was highest after an ICrH, mostly due to deaths caused by ICH. There was a vast difference in mortality between patients with different functional levels at discharge. A serious hemorrhage was associated with increased mortality in patients with high functional levels, but it did not increase the already high mortality during follow-up among patients with low functional status. A diagnosis of hypertension prior to the IS/TIA was associated with an increased risk of serious hemorrhage.

## Supporting information

S1 Table ICD-10 codes for hemorrhage diagnosis. (DOCX)

S2 Table Modified rankin scale. (DOCX)

S3 Table Background data on patients that were hospitalized for an ischemic stroke or transient ischemic attack. (DOCX)

S4 Table Thirty-day case fatality after a serious hemorrhage among patients hospitalized with ischemic stroke or transient ischemic attack and with different functional status at discharge. (DOCX)

S5 Table Univariable and multivariable results indicate significant (bold) risk factors for hemorrhage after hospitalization for an ischemic stroke or transient ischemic attack. (DOCX)

S1 Fig Flow chart. (DOCX)

S2 Fig Flow chart. (DOCX)
