# Supplementary material for: Serious hemorrhages after ischemic stroke or TIA – Incidence, mortality, and predictors
Source: PLoS One. 2018 Apr 5;13(4):e0195324. doi: 10.1371/journal.pone.0195324 (PMC5886551; doi:10.1371/journal.pone.0195324)
Supplement: S1 Table — (DOCX) [file pone.0195324.s001.docx]

**S1 Table.** **ICD-10 codes for hemorrhage diagnosis**

- D62.9 – Acute posthemorrhagic anemia
- D50.0 – Iron deficiency anemia secondary to blood loss
- H11.3 – Conjunctival hemorrhage
- H31.3 – Choroidal hemorrhage
- H35.6 – Retinal hemorrhage
- H43.1, H45.0 – Vitreous hemorrhage
- H92.2 – Hemorrhage from the ear
- I60 – Subarachnoidal hemorrhage
- I61 – Intracerebral hemorrhage
- I62 – Non traumatic intracranial hemorrhage
- I 69.0, I69.1, I69.2 – Sequel of intracranial hemorrhage
- I84.1 I84.4, I84.8 – Hemorrhoids
- I85.0, I98.3 – Esophageal varices
- K22.6 – Gastro-esophageal laceration hemorrhage
- K25 - 28 – Acute peptic ulcer
- K29 - Acute hemorrhagic gastritis
- K62.5 – Hemorrhage of anus and rectum
- K92.0 – Hematemesis
- K92.1 – Melena
- K92.2 – Gastrointestinal hemorrhage, unspecified
- M25.0 – Hemarthrosis
- N42.1 – Congestion and hemorrhage of prostate
- N93.8, N93.9 – Abnormal uterine and vaginal bleeding
- N95.0 – Postmenopausal hemorrhage
- R04.1, R04.2, R04.8, R04.9 – Hemorrhage from throat and airways
- R31.9 – Hematuria
- S06.4, S06.5, S06.6 – Epidural hemorrhage, traumatic subarachnoidal and subdural
- R58.9 – Unspecified hemorrhage
- T81.0 – Hemorrhage as complication to surgery
