# Supplementary material for: Serious hemorrhages after ischemic stroke or TIA – Incidence, mortality, and predictors
Source: PLoS One. 2018 Apr 5;13(4):e0195324. doi: 10.1371/journal.pone.0195324 (PMC5886551; doi:10.1371/journal.pone.0195324)
Supplement: S2 Table — (DOCX) [file pone.0195324.s002.docx]

**S2 Table.** **Modified Rankin Scale**

0 - No symptoms at all

1 - No significant disability despite symptoms: able to carry

out all usual duties and activities

2 - Slight disability: unable to carry out all previous activities,

but able to look after own affairs without assistance

3 - Moderate disability: requiring some help, but able to walk

without assistance

4 - Moderately severe disability: unable to walk without assistance,

and unable to attend to own bodily needs

without assistance

5 - Severe disability: bedridden, incontinent, and requiring

constant nursing care and attention
