# Supplementary material for: Serious hemorrhages after ischemic stroke or TIA – Incidence, mortality, and predictors
Source: PLoS One. 2018 Apr 5;13(4):e0195324. doi: 10.1371/journal.pone.0195324 (PMC5886551; doi:10.1371/journal.pone.0195324)
Supplement: S3 Table — (DOCX) [file pone.0195324.s003.docx]

**S3 Table.** **Background data on patients that were hospitalized for an ischemic stroke or transient ischemic attack**

|  | All | mRS at discharge | |
| --- | --- | --- | --- |
|  |  | 0-2 | 3-5 |
| All patients, N | 1528 | 1002 | 526 |
| Female | 681 (44.6%) | 414 (41.3%) | 267 (50.8%) |
| Age, y (mean) | 75.1 | 72.1 | 80.8 |
| Smoker | 191 (12.7%) | 149 (14.9%) | 42 (8.4%) |
| GFR at index event, mL/min/1.73 m^2^ (mean) | 74 | 80 | 61 |
| Ischemic stroke as index event | 1083 (70.9%) | 596 (59.5%) | 487 (92.6%) |
| Diagnosis prior to IS or TIA |  |  |  |
| Hypertension | 985 (64.5%) | 612 (61.1%) | 373 (70.9%) |
| Myocardial Infarction | 182 (11.9%) | 103 (10.3%) | 79 (15.0%) |
| Heart Failure | 123 (8.0%) | 49 (4.9%) | 74 (14.1%) |
| Ischemic stroke | 235 (15.4%) | 111 (11.1%) | 124 (23.6%) |
| ICrH | 39 (2.6%) | 14 (1.4%) | 25 (4.8%) |
| GI hemorrhage | 104 (6.8%) | 55 (5.5%) | 49 (9.3%) |
| Diabetes at discharge | 305 (20.0%) | 174 (17.4%) | 131 (24.9%) |
| Atrial fibrillation | 391 (25.6%) | 200 (20.0%) | 191 (36.3%) |
| Treatment at discharge |  |  |  |
| Statin | 951 (62.2%) | 712 (71.1%) | 239 (45.4%) |
| Antihypertensive | 1184 (77.5%) | 762 (76.0%) | 422 (80.2%) |
| Anticoagulant | 244 (16.0%) | 167 (16.7%) | 77 (14.6%) |
| Antiplatelet | 1233 (80.7%) | 822 (82.0%) | 411 (78.1%) |
| CHA2DS2-Vasc at discharge (mean) | 4.9 | 4.6 | 5.4 |
| mRS at discharge (mean) | 1.8 | 0.8 | 3.7 |

Values represent percentage of patients in each group, unless otherwise indicated.

Abbreviations: GFR indicates glomerular filtration rate; IS, ischemic stroke; TIA, transitoric ischemic attack; ICrH, intracranial hemorrhage; GI, gastrointestinal and mRS, modified Rankin scale.
