# Supplementary material for: Serious hemorrhages after ischemic stroke or TIA – Incidence, mortality, and predictors
Source: PLoS One. 2018 Apr 5;13(4):e0195324. doi: 10.1371/journal.pone.0195324 (PMC5886551; doi:10.1371/journal.pone.0195324)
Supplement: S4 Table — (DOCX) [file pone.0195324.s004.docx]

**S4 Table. Thirty-day case fatality after a serious hemorrhage among patients hospitalized with ischemic stroke or transient ischemic attack and with different functional status at discharge**

| Condition | All | | mRS 0-2 | | mRS 3-5 | |
| --- | --- | --- | --- | --- | --- | --- |
|  | n | % | n | % | n | % |
| All hemorrhages | 18/113 | 15.9 | 11/74 | 14.9 | 7/39 | 18.0 |
| ICrH | 11/45 | 24.4 | 8/32 | 25.0 | 3/13 | 23.1 |
| ICH | 8/19 | 42.1 | 5/13 | 38.5 | 3/6 | 50.0 |
| GI hemorrhages | 5/41 | 12.2 | 2/23 | 8.7 | 3/18 | 16.7 |
| Others | 2/27 | 7.4 | 1/19 | 5.3 | 1/8 | 12.5 |

Values represent number of patients who died within 30 days of all patients with a serious hemorrhage in each category (n) and percentage of patients in each group (%).

ICrH indicates intracranial hemorrhage; ICH, intracerebral hemorrhage; GI gastrointestinal and mRS, modified Rankin scale.
