# Supplementary material for: Serious hemorrhages after ischemic stroke or TIA – Incidence, mortality, and predictors
Source: PLoS One. 2018 Apr 5;13(4):e0195324. doi: 10.1371/journal.pone.0195324 (PMC5886551; doi:10.1371/journal.pone.0195324)
Supplement: S5 Table — (DOCX) [file pone.0195324.s005.docx]

**S5 Table.** **Univariable and multivariable results indicate significant (bold) risk factors for hemorrhage after hospitalization for an ischemic stroke or transient ischemic attack**

|  | Univariable | | Multivariable 1 | | Multivariable 2 | |
| --- | --- | --- | --- | --- | --- | --- |
|  | HR (95% CI) | p-value | HR (95% CI) | p-value | HR (95% CI) | p-value |
| Age ≥75 years | 1.86 (1.26-2.74) | **0.002** | 1.37 (0.87-2.14) | 0.173 | 1.59 (1.06-2.39) | **0.026** |
| Women | 0.83 (0.57-1.20) | 0.317 | 0.70 (0.47-1.03) | 0.068 | 0.73 (0.50-1.07) | 0.111 |
| BMI ≥30 | 0.73 (0.44-1.23) | 0.237 |  |  |  |  |
| Smoker | 0.75 (0.41-1.37) | 0.350 |  |  |  |  |
| GFR at admission ≤60 mL/min/1.73 m^2^ | 1.78 (1.23-2.58) | **0.002** | 1.38 (0.90-2.11) | 0.136 |  |  |
| Ischemic stroke (compared to TIA) | 1.36 (0.89-2.07) | 0.155 |  |  |  |  |
| Prior Hypertension | 1.90 (1.23-2.92) | **0.004** | 1.73 (1.12-2.68) | **0.014** | 1.79 (1.16-2.77) | **0.009** |
| Prior Myocardial infarction | 1.40 (0.84-2.34) | 0.202 |  |  |  |  |
| Prior Heart failure | 2.62 (0.83-8.28) | 0.101 |  |  |  |  |
| Prior Ischemic stroke or TIA | 1.18 (0.78-1.80) | 0.439 |  |  |  |  |
| Prior diagnosis of ICrH | 1.69 (0.62-4.60) | 0.301 |  |  |  |  |
| Prior diagnosis of GI hemorrhage | 1.67 (0.89-3.10) | 0.108 |  |  |  |  |
| Diabetes at discharge | 1.00 (0.63-1.60) | 0.997 |  |  |  |  |
| Statin at discharge | 0.59 (0.40-0.85) | **0.005** | 0.66 (0.44-0.97) | **0.036** | 0.63 (0.43-0.93) | **0.019** |
| Anticoagulant at discharge | 1.12 (0.69-1.81) | 0.658 |  |  |  |  |
| Antiplatelet at discharge | 0.80 (0.51-1.26) | 0.340 |  |  |  |  |
| mRS 3-6 at discharge | 1.46 (0.99-2.15) | **0.057** | 1.17 (0. -1.75) | 0.460 |  |  |
| CHADS-Vasc 4-8 | 4.81 (2.11-10.94) | **0.000** |  |  |  |  |
| Atrial fibrillation | 1.26 (0.84-1.89) | 0.263 |  |  |  |  |

Values represent percentage of patients in each group, unless otherwise indicated. Multivariable 1 includes factors with p<0.100 in the univariable analysis and Multivariable 2 includes factors with p<0.100 in Multivariable 1 analysis. Both Multivariable 1 and 2 also includes age and sex.

Abbreviations: BMI indicates body mass index; GFR, glomerular filtration rate; TIA, transitoric ischemic attack; ICrH, intracranial hemorrhage; GI, gastrointestinal; HR, hazard ratio and CI, confidence interval.
