# Supplementary material for: Serious hemorrhages after ischemic stroke or TIA – Incidence, mortality, and predictors
Source: PLoS One. 2018 Apr 5;13(4):e0195324. doi: 10.1371/journal.pone.0195324 (PMC5886551; doi:10.1371/journal.pone.0195324)
Supplement: S2 Fig — (DOCX) [file pone.0195324.s007.docx]

**S2 Figure. Flow chart**
